# Metabolic engineering of the fungal D-galacturonate pathway for L-ascorbic acid production

**DOI:** 10.1186/s12934-014-0184-2

**Published:** 2015-01-08

**Authors:** Joosu Kuivanen, Merja Penttilä, Peter Richard

**Affiliations:** VTT Technical Research Centre of Finland, PO Box 1000, 02044 VTT Espoo, Finland

**Keywords:** L-ascorbic acid, D-galacturonic acid, L-galactonic acid, Citrus peel, *Aspergillus niger*, Metabolic engineering

## Abstract

**Background:**

Synthetic L-ascorbic acid (vitamin C) is widely used as a preservative and nutrient in food and pharmaceutical industries. In the current production method, D-glucose is converted to L-ascorbic acid via several biochemical and chemical steps. The main source of L-ascorbic acid in human nutrition is plants. Several alternative metabolic pathways for L-ascorbic acid biosynthesis are known in plants. In one of them, D-galacturonic acid is the precursor. D-Galacturonic acid is also the main monomer in pectin, a plant cell wall polysaccharide. Pectin is abundant in biomass and is readily available from several waste streams from fruit and sugar processing industries.

**Results:**

In the present work, we engineered the filamentous fungus *Aspergillus niger* for the conversion of D-galacturonic acid to L-ascorbic acid. In the generated pathway, the native D-galacturonate reductase activity was utilized while the gene coding for the second enzyme in the fungal D-galacturonic acid pathway, an L-galactonate consuming dehydratase, was deleted. Two heterologous genes coding for enzymes from the plant L-ascorbic acid pathway – L-galactono-1,4-lactone lactonase from *Euglena gracilis* (*EgALase*) and L-galactono-1,4-lactone dehydrogenase from *Malpighia glabra* (*MgGALDH*) – were introduced into the *A. niger* strain. Alternatively, an unspecific L-gulono-1,4-lactone lactonase (*smp30*) from the animal L-ascorbic acid pathway was introduced in the fungal strain instead of the plant L-galactono-1,4-lactone lactonase. In addition, a strain with the production pathway inducible with D-galacturonic acid was generated by using a bidirectional and D-galacturonic acid inducible promoter from the fungus. Even though, the lactonase enzyme activity was not observed in the resulting strains, they were capable of producing L-ascorbic acid from pure D-galacturonic acid or pectin-rich biomass in a consolidated bioprocess. Product titers up to 170 mg/l were achieved.

**Conclusions:**

In the current study, an L-ascorbic acid pathway using D-galacturonic acid as a precursor was introduced to a microorganism for the first time. This is also the first report on an engineered filamentous fungus for L-ascorbic acid production and a proof-of-concept of consolidated bioprocess for the production.

## Background

L-Ascorbic acid (L-AA), also known as vitamin C, is a six-carbon organic compound with reducing agent properties. It occurs naturally in many animal and plant cells having biological functions, such as being an antioxidant and enzyme cofactor [1,2]. Synthetic L-AA is commercially used for several purposes in food, beverage, feed and pharmaceutical industries. The annual production of synthetic L-AA is about 110 000 tonnes with the fluctuating market price of about 10 USD per kg [3].

Industrial L-AA production has been traditionally based on the Reichstein process which is an efficient multi-step and mostly chemical manufacturing method converting D-glucose to L-AA [4]. In the first step, D-glucose is hydrogenated to D-sorbitol followed by the oxidation of D-sorbitol to L-sorbose that is commonly carried out using a bacterial fermentation [3]. The resulting L-sorbose is then chemically oxidized to 2-keto-L-gulonate and lactonized to L-AA. Other proposed and reported biotechnological steps have focused on the microbial conversion of D-sorbitol, L-sorbose or D-glucose to 2-keto-L-gulonic acid that is the last intermediate in the Reichstein process [5]. Currently, the predominant industrial process for L-AA production is a so called two-step fermentation process [3]. In the first step, D-sorbitol is oxidized to L-sorbose using *Gluconobacter oxydans*. The second step is a mixed fermentation with *Ketogulonicigenium vulgare* and *Bacillus megaterium*, converting L-sorbose to 2-keto-L-gulonic acid which is then chemically converted to L-AA.

In addition to the partially biotechnological processes, some approaches using wild type or engineered microbes for the direct L-AA production have been reported in the literature. A one-step process from biomass sugar to L-AA would be advantageous when compared to the currently used two-step fermentation process. Yeast have a native biosynthetic pathway for D-erythroascorbic acid (D-EA), a 5-carbon analogue of L-AA, where D-arabinose is converted to D-EA via D-arabino-1,4-lactone [6]. The D-EA pathway seems to be relatively unspecific converting also L-galactose and L-galactono-1,4-lactone (L-galL) to L-AA [7,8]. The reactions are similar to the reactions of L-AA synthesis in plants via the Smirnoff-Wheeler (S-W) pathway. In addition to yeast, a strain of *K. vulgare*, the bacterium that is used in the two-step fermentation process, is capable of converting D-sorbitol, L-sorbose, L-sorbosone and L-gulose directly to L-AA [9]. Also several strains of algae producing small concentrations of extracellular L-AA have been reported in the literature [10].

In the field of metabolic engineering, there are a few attempts to introduce the plant S-W pathway to yeast either by overexpressing the native substrate-unspecific D-EA pathway genes or expressing the S-W pathway genes from *Arabidopsis thaliana*. The resulting strains were capable of converting L-galactose, D-glucose and D-galactose to L-AA [11–13]. The product titers were reported to be about 100 mg l^−1^ from L-galactose [11], 0.1 mg l^−1^ OD^−1^ from D-glucose [12] and 30 mg l^−1^ from D-galactose [13].

In plants, L-AA is considered to be synthesized predominantly through the S-W pathway where D-glucose is converted via GPD-D-mannose and L-galactose to L-galL which is oxidized to L-AA in the final step by the L-galL dehydrogenase (GALDH) [14]. In addition to S-W, a few alternative pathways for L-AA synthesis have been observed in plants. One of them originates from D-galacturonic acid (D-galUA) which is reduced to L-galactonic acid (L-galA) and further lactonized to L-galL, which is the last intermediate in the S-W pathway [1]. Even though the function of a D-galUA reductase for L-AA biosynthesis was shown in higher plants [15], the evidence of the pathway converting D-galUA to L-AA is still incomplete – the gene coding for an L-galL aldonolactonase (ALase) catalysing the lactonization of L-galA to L-galL has been described only in the photosynthetic organism *Euglina gracilis* [16]. This activity was however found in animals as part of the biosynthetic L-AA pathway. In the animal pathway, L-gulonic acid is lactonized to its corresponding 1,4-lactone by an ALase that is encoded by the *smp30* gene. The enzyme is unspecific and can also catalyse the lactonization of L-galA [17].

**Figure 1:**
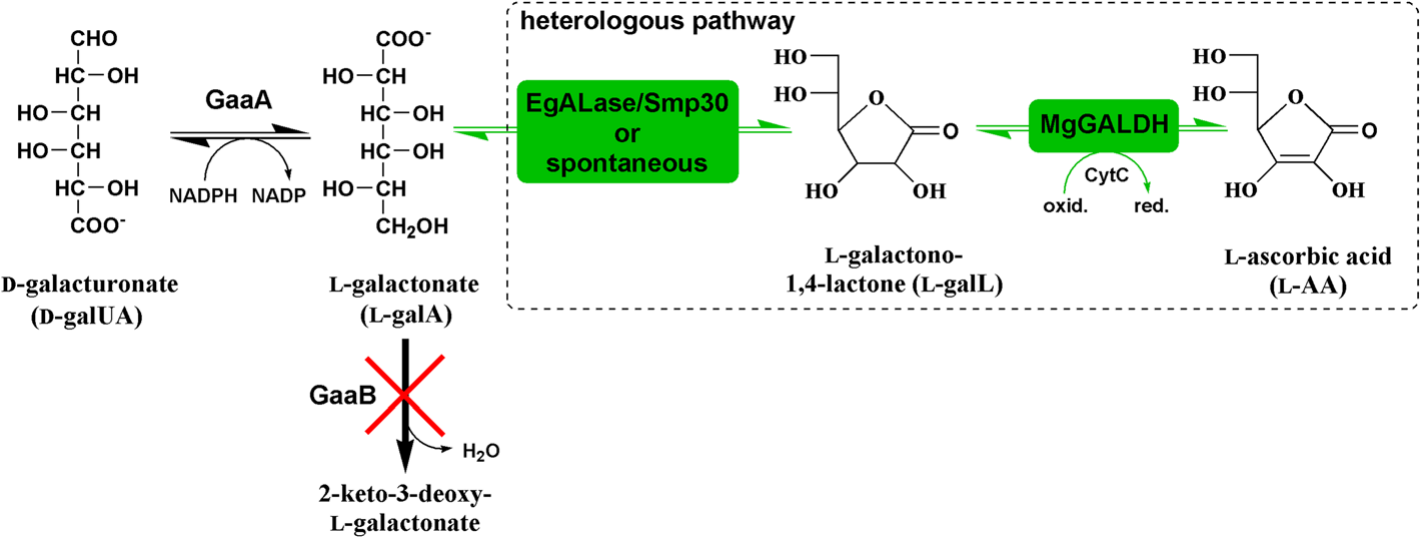
**Schematic figure of the disrupted fungal D-galUA pathway and the heterologous pathway introduced in** ***A. niger*** **for L-AA biosynthesis: native GaaA (D-galUA reductase), native GaaB (L-galA dehydratase), EgALase (L-galL aldonolactonase from** ***Euglena gracilis*** **), Smp30 (aldonolactonase from rat functioning in the animal L-AA pathway) and MgGALDH (L-galL dehydrogenase from Malpighia glabra).**

In the present study, instead of using the plant S-W pathway for L-AA synthesis, we have focused on the alternative pathway converting D-galUA to L-AA. D-GalUA is the main monomer in pectin, a heteropolysaccharide found from plant cell walls, which is especially abundant in some non-woody biomass types, such as citrus fruit peels and sugar beet pulp. Both of these materials are abundantly available as agro-industrial side streams. The enzymes for degradation of pectin to D-galUA the catabolic pathways for its utilization are known in many microbial organisms. In fungi, such as *Aspergillus niger*, the pathway for D-galUA catabolism is reductive having a similar reduction step from D-galUA to L-galA as in the L-AA synthesis pathway originating from D-galUA in plants (Figure 1) [18]. Previously, we reported engineered *A. niger* strains capable of converting pure D-galUA and citrus processing waste (CPW) to L-galA that can be chemically converted to L-AA [19,20]. In the present study, we have engineered *A. niger* strains that are capable of direct production of L-AA from D-galUA and CPW.

## Results

### Pathway assembly and transcription

The fungal catabolic D-galUA pathway and the proposed D-galUA originating biosynthetic L-AA pathway in plants share the same reduction step yielding L-galA in the first reaction (Figure 1). Thus, the *A. niger* strain Δ*gaaB* with the disrupted L-galA dehydratase gene, which is known to accumulate L-galA, was used as a platform strain. In addition, two heterologous genes from the plant or animal L-AA pathway, an ALase and a GALDH, were introduced to the strain. Several different *A. niger* Δ*gaaB* based strains with an ALase (*EgALase* or *smp30*) and a GALDH (*MgGALDH*) or only the GALDH were generated and tested (Table 1). The *MgGALDH* gene that was used contained its native mitochondrial signal peptide region at the beginning of the ORF. In addition, a constitutive (*PgpdA*) and a D-galUA inducible (*PgaaA*/*C*) promoter were compared.

**Table 1:** **Plasmids and** ***A. niger*** **strains used in this work**

**Plasmid**	**Genes expressed**	**Promoter**
JKp1-EgALase	The codon optimized *Euglina gracilis* Alase (*EgALase*) [GenBank: AB306917]	*gpdA*
JKp1-Smp30	The codon optimized rat *Smp30* [GenBank: CAA48786]	*gpdA*
JKp1-MgGALDH	The codon optimized *Malpighia glabra* GALDH (*MgGALDH*) [GenBank: ACG75919] ORF	*gpdA*
Bidir-EgALase-MgGALDH	*EgALase* and *MgGALDH*	*gaaA/C*
**Strain**	**Genes expressed**	**Description**
*∆gaaB*	none	ATCC 1015 with the deleted L-GalA dehydratase gene gaaB
*∆gaaB-Mg*	*MgGALDH*	*∆gaaB* with *MgGALDH* expressed under *gpdA* promoter
*∆gaaB-Eg*-*Mg* (*PgpdA*)	*EgALase* and *MgGALDH*	*∆gaaB* with *EgALase* and *MgGALDH*, both expressed under *gpdA* promoter
*∆gaaB-Smp30*-*Mg*	*Smp30* and *MgGALDH*	*∆gaaB* with *Smp30* and *MgGALDH*, both expressed under *gpdA* promoter
*∆gaaB-Eg*-*Mg* (*PgaaA/C*)	*EgALase* and *MgGALDH*	*∆gaaB* with *EgALase* and *MgGALDH*, both expressed under bidirectional *gaaA/C* promoter

**Figure 2:**
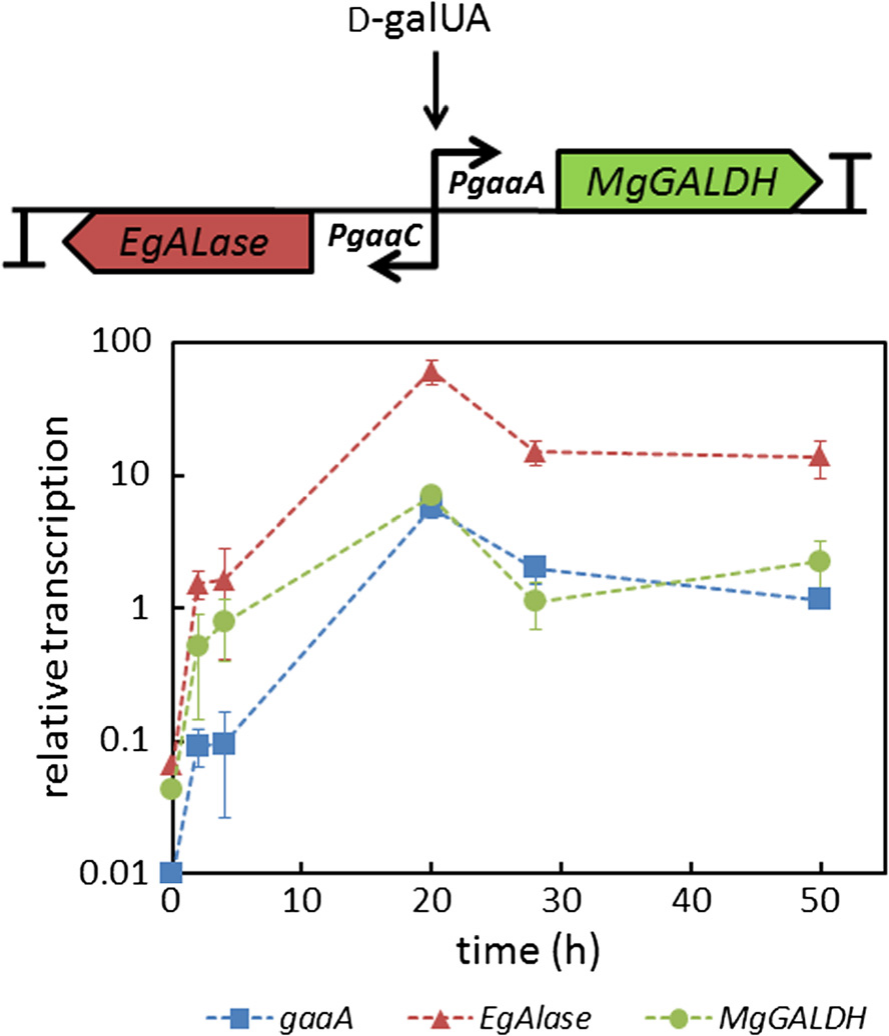
**The D-galUA inducible expression cassette and relative transcription of** ***gaaA*** **(blue squares),** ***EgALase*** **(red triangles) and** ***MgGALDH*** **(green circles) in the strain Δ** ***gaaB*** **-** ***Eg*** **-** ***Mg*** **(** ***PgaaA/C*** **) after the shift from pre-cultures to medium supplemented with D-galUA and D-xylose.** Values were normalized to the expression of actin. Error bars represent ± SEM (n = 3).

To avoid unnecessary and energy wasting expression of the L-AA pathway genes in non-producing conditions, the strain Δ*gaaB-Eg-Mg* (*PgaaA/C*) with D-galUA inducible *EgALase* and *MgGALDH* expression was generated using the bidirectional *gaaA*/*C* promoter. Since there are no reports on the use of *gaaA/C* promoter in heterologous expression, the functionality of the D-galUA inducible transcription of *EgALase* and *MgGALDH* from the *gaaA/C* promoter was tested using RT-qPCR (Figure 2). The transcription of both of the heterologous genes was clearly induced at 20 hours after the shift to D-galUA containing medium. The pattern of *MgGALDH* transcription was similar to the transcription of the native *gaaA* gene, which was expected since *MgGALDH* was orientated in the same way behind the bidirectional *gaaA/C* promoter as the *gaaA* gene is. The transcription of *EgALase* was higher when compared to *gaaA* and *MgGALDH* after the D-galUA induction. A similar observation was reported earlier in the case of native *gaaC* gene (2-keto-3-deoxy-L-galactonate aldolase) which had a higher transcription when compared to *gaaA* growing in D-galUA medium [21]. *EgALase* had the same orientation at *gaaA/C* promoter as *gaaC* gene in the wild type strain.

### Enzyme activities

**Figure 3:**
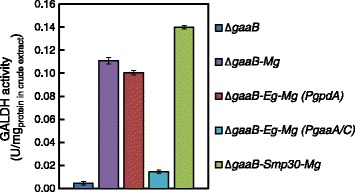
**GALDH activity for L-galL from the crude extracts of Δ** ***gaaB*** **, Δ** ***gaaB*** **-** ***Mg*** **Δ** ***gaaB*** **-** ***Eg*** **-** ***Mg*** **(** ***PgpdA*** **), Δ** ***gaaB*** **-** ***Eg*** **-** ***Mg*** **(** ***PgaaA/C*** **) and Δ** ***gaaB*** **-** ***Smp30*** **-** ***Mg*** **cultured in minimal medium supplemented with D-galUA and D-xylose for 20 h.** All of the values from the strains with introduced *MgGALDH* differed significantly from the value from Δ*gaaB* (P < 0.05, Student’s *t*-test). Error bars represent ± SEM (n = 3).

Enzyme activities of the introduced L-AA pathway genes were measured from crude extracts. The GALDH activity assay was based on the measurement of reduced cytochrome C as a result from oxidation of L-galL. The activity was tested from the parental strain Δ*gaaB* and from the further engineered strains that were cultured 20 h in the medium supplemented with 20 g l^−1^ D-galUA and 5 g l^−1^ D-xylose (Figure 3). All the strains with *MgGALDH* under the constitutive *gpdA* promoter showed the activity for L-galL while only residual activity was found from the parental strain Δ*gaaB*. In the case of Δ*gaaB*-*Eg*-*Mg* (*PgaaA/C*) strain, the GALDH activity was lower; however, it was still significantly higher if compared to the values from Δ*gaaB* (P < 0.05, Student’s *t*-test).

In the lactonization of L-galA to L-galL, which is catalysed by an ALase, protons are absorbed in an equimolar manner increasing the pH in the reaction. The relation between absorption at 405 nm and proton concentration was quantified being ∆A_405_ = 0.003 per 10 μM of protons which should provide high enough sensitivity for the ALase assay. We tested all the engineered strains for ALase activity in the presence of L-galA; however, we could not detect this activity.

### Production of L-AA

**Figure 4:**
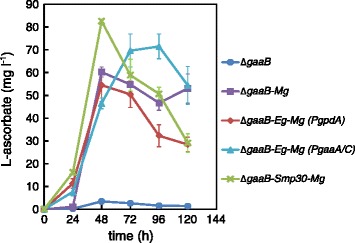
**L-Ascorbate production by engineered** ***A. niger*** **strains in minimal medium supplemented with D-galUA and D-xylose.** Error bars represent ± SEM (n = 3).

The parental strain Δ*gaaB* and the different engineered strains were tested for L-AA production even though the lactonase activities were not detectable. We used flask cultures on defined medium supplemented with D-galUA and D-xylose and with the initial pH adjusted to 3.0. With the strain Δ*gaaB* only L-AA concentrations of below 3.5 mg l^−1^ were observed in the cultures (Figure 4). All the engineered strains having *MgGALDH* under *gpdA* or *gaaA*/*C* promoter produced L-AA at concentrations of around 55–83 mg l^−1^. Also the strain Δ*gaaB*-*Mg* without an ALase produced similar L-AA concentrations as the strain Δ*gaaB*-*Eg*-*Mg* (*PgpdA*) with the introduced *EgALase* gene. In fact, the L-AA production corresponds to the GALDH activity in the strains having *MgGALDH* under *PgpdA* (Figure 3). The clearest difference between the engineered strains was the timing of the highest L-AA concentration in the medium – all the strains with *MgGALDH* under *gpdA* promoter had the highest titer at 48 h whereas Δ*gaaB*-*Eg*-*Mg* (*PgaaA/C*) had the highest titer at 96 h. The decreasing L-AA concentrations during the time course may be due to the instability of the product causing by the exposure to air. In addition, the production turned out to be pH dependent since L-AA production was not observed when the initial pH was 5.0 (data not shown).

**Figure 5:**
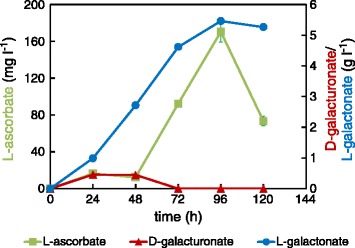
**Concentration of extracellular L-ascorbate (green squares), D-galUA (red triangles) and L-galA (blue circles) from a culture of the strain Δ** ***gaaB*** **-** ***Eg*** **-** ***Mg*** **(** ***PgaaA/C*** **) in minimal medium supplemented with CPW.** Error bars represent ± SEM (n = 3) and where not visible, are smaller than the symbol.

Even though, D-xylose was used as a co-substrate, one of the reasons for limited L-AA production may be the lack of NADPH for the D-galUA reductase *gaaA* in the first reaction in the pathway. In addition, energy and carbon sources are needed for the expression of heterologous L-AA pathway genes. To overcome these possible limitations and to test L-AA production from a pectin-rich residue, we used CPW as substrate containing a complex mix of different sugars and sugar acids in the form of polysaccharides. We decided to use Δ*gaaB*-*Eg*-*Mg* (*PgaaA/C*) strain in order to avoid unnecessary and energy wasting expression of the heterologous genes at the beginning of fermentation when free D-galUA is not yet available. In addition, we used higher fungal biomass in the inoculation in order to ensure CPW hydrolysis and speed up the process. L-AA production from CPW turned out to be more efficient than in defined media conditions with respect to the final concentrations (Figure 5). A lag phase of about 48 h was observed before the production started at a higher rate, which is probably due to rather slow pectin hydrolysis to D-galUA and utilization of the more preferred carbon sources, such as D-glucose present in CPW. The D-galUA content in CPW that was prepared as the CPW used in this study was reported to be 27% on a dry mass basis [20]. Other detected main components after pectinase hydrolysis were L-arabinose, D-galactose, D-glucose and L-rhamnose. Even though, L-AA production started later than when compared to the defined conditions with the Δ*gaaB*-*Eg*-*Mg* (*PgaaA/C*) strain the highest titer (around 170 mg l^−1^) was achieved at 96 h as was the case of the cultures in the defined conditions. As in the case of defined conditions, a decrease in L-AA concentration was observed after 96 h probably due to the instability of the product. The CPW used as a substrate did not contain detectable amounts of L-AA. In the control fermentations with the parental strain Δ*gaaB*, the observed L-AA concentrations were below 15 mg l^−1^.

## Discussion

Synthetic L-AA is a widely used compound in several industries. Currently it is produced in a multistep process including chemical and biochemical steps. This study is the first report on an engineered filamentous fungus for direct L-AA production. To the best of our knowledge, the L-AA titer of 170 mg l^−1^ that was achieved in this work is the highest among engineered fungal strains. In addition, the pathway for L-AA biosynthesis originating from D-galUA was for the first time introduced to a heterologous host. In the previous literature, L-AA production was reported to be achievable from D-glucuronic acid and D-galUA using wild type strains of *A. niger* [22] or yeast [23]. However, the detection method for L-AA that was used in these studies (2,6-dichlorophenolindophenol method) could not distinguish between L-AA and other similar compounds, such as D-EA [24].

We decided to use the GALDH from *M. glabra* (acerola), which is known for its vitamin C rich fruits, including the native N-terminal mitochondrial signal peptide. The kinetic parameters of MgGALDH are not known but the expression level of *MgGALDH* is known to be higher in *M. glabra* tissue when compared to the corresponding GALDH gene (*AtGALDH*) expression in *Arabidopsis thaliana* [25]. *AtGALDH* gene codes for a protein having 75.25% homology with MgGALDH and kinetic parameters of K_m_ 0.17 mM and k_cat_ 134 s^−1^ for L-galL [26]. We have also tested the expression of *AtGALDH* gene in the *A. niger* Δ*gaaB* strain, however, L-AA was not observed in the resulting strains (data not shown). In plants, the GALDH locates on the inner membrane of mitochondria binding non-covalently FAD as a cofactor [26]. During the oxidation of L-galL to L-AA, electrons are shuttled to cytochrome c and further to the electron transport chain. The recombinant AtGALDH without mitochondrial targeting signal has been produced in *E. coli* as a cytoplasmic protein [26]. The resulting enzyme was active with L-galL when cytochrome c was added to the reaction mixture. However, in a living cell the functionality of a GALDH is probably dependent on its correct localization enabling interaction with mitochondrial cytochrome c. Thus it is crucial that the mitochondrial targeting signal in a plant GALDH is also functional in *A. niger*. Mitochondrial inner membrane proteins are most often targeted to their location by an N-terminal cleavable peptide [27]. The targeting peptide is typically positively charged among different organisms. However, in yeast, mitochondrial signal peptides are generally shorter, less alpha-helix forming and less hydrophobic when compared with the plant signal peptides [27]. The cleavage site for the signal peptides seem to be relatively conserved between yeast and plants consisting of tyrosine, leucine, phenylalanine and arginine residues [27]. In the case of *AtGALDH* and *MgGALDH*, the estimated signal peptides differ being 102 and 84 residues long, respectively [28]. The shorter signal peptide in *MgGALDH* may be more suitable for mitochondrial targeting in *A. niger*, however, the localization of MgGALDH in the engineered *A. niger* strains was not investigated in this study.

Despite several attempts, the ALase activity for L-galL could not be detected from any of *EgALase* or *smp30* expressing strains. In addition to *A. niger* strains, we have also tried to express both of the ALases in yeast or *E. coli* strains, however, without detectable activity. The lactonase assay that was used in this work is based on a pH change and a pH indicator in the reaction mixture. The assay should provide high enough sensitivity for the activity and it has been routinely and successfully used in many studies. Consequently, the most likely explanations for the non-detectable ALase activity are inactivity of the protein in heterologous hosts or inactivation during cell disruption. In the literature, EgALase was produced at low temperature (15°C) in an *E. coli* strain co-expressing the Trigger Factor chaperone protein [16]. In the case of Smp30 from animal L-AA pathway, the recombinant protein was produced in *E. coli* co-expressing two chaperones GroEL and GroES [17]. In addition, it has been reported that production of human Smp30 protein in *E. coli* without chaperones led up to the insoluble and inactive proteins and formation of inclusion bodies [29,30]. Thus poor folding of the ALase proteins in a heterologous host is a possible explanation for the unsuccessful expression.

Regardless of the non-detectable ALase activity, several *A. niger* strains with a functional GALDH were capable of L-AA production. The lactonization reaction might be spontaneous and may take place extracellularly. In the lactonization reaction, protons are assimilated, which mean that a low pH shifts the equilibrium towards the lactone form. This would explain the fact that only low pH enabled L-AA production. Thus it is possible that L-galA is first secreted out from the cells and spontaneously formed L-galL is then transported back to the cells and oxidized to L-AA. On the other hand, the parental strain *∆gaaB* is known for its higher capacity to produce L-galA at low pH [19,20] which may provide more substrate for the L-AA pathway also in the engineered strains when cultured at low pH. The production improved when CPW was used as substrate. In addition to the higher fungal biomass in the inoculation, the improvement was possibly due to the additional carbon sources that were constantly released from the substrate during the fermentation providing energy such as reducing power for the strain. The product titer was still relatively low and possible limited by the poor lactonization reaction from L-galA to L-galL. In addition, L-AA concentrations started to decrease during the fermentation. This is likely due to the oxidation that occurs by the exposure to air. Thus it would be beneficial for the process to optimize factors, such as, aeration and timing of the harvesting. Nevertheless, this is the first demonstration of a consolidated bioprocess for L-AA production.

## Conclusions

We have engineered *A. niger* strains to redirect the D-galUA pathway to L-AA synthesis. The native catabolic D-galUA pathway was disrupted and the biosynthetic L-AA pathway from plants originating from D-galUA was introduced. In addition, we built a strain having the L-AA pathway under D-galUA inducible expression. The resulting strains were capable of L-AA production from pure D-galUA but also from CPW that is a pectin-rich biomass residue. Final L-AA titers up to around 170 mg l^−1^ were achieved with the engineered strains.

## Methods

### Strains

The *Aspergillus niger* strain ATCC 1015 (CBS 113.46) was used as a wild type. The engineered strain of ATCC 1015 with the deleted L-galA dehydratase *gaaB* [19,20] was used as a host strain for the L-AA production strains that are listed in Table 1. All the plasmids were produced in *E. coli* TOP10 cells and the homologous recombination for the plasmid construction was carried out in the *S. cerevisiae* strain ATCC 90845.

### Plasmid construction

For introducing the biosynthetic L-AA pathway in *A. niger*, the heterologous genes coding for a GALDH or an ALase, listed in Table 1, were custom synthetized as codon optimized ORFs (Genscript) and inserted to the JKp1-hph plasmid [19] under the *gpdA* promoter. Alternatively, the GALDH from *Malpighia glabra* (*MgGALDH*) and the ALase from *Euglena gracilis* (*EgALase*) were both expressed under the native bidirectional promoter (657 bp) of the *A. niger* D-galUA reductase (*gaaA*) and the 2-keto-3-deoxy-L-galactonate aldolase (*gaaC*) genes that are clustered in the genome. In the expression cassette, *MgGALDH* was orientated as *gaaA* and *EgALase* as *gaaC* in *A. niger* genome. Terminators that were used in the bidirectional cassette for *MgGALD*H and *EgALase* were the native *gaaA* and *gaaC* terminators, respectively, both 505 bp downstream from the stop codon. All of the expression cassettes contained hygromycin B phosphotransferase (*hph*) gene under the *gpdA* promoter for the selection of transformants.

The codon optimized ORFs were released with *SacI* and *XmaI* (both NEB) and ligated to the JKp1 plasmid. The resulting plasmids JKp1-EgALase/Smp30/MgGALDH were linearized with *SpeI* (NEB). For constructing the cassette with D-galUA inducible expression, the bidirectional *gaaA*/*C* promoter and terminators of *gaaA* and *gaaC* genes were amplified from *A. niger* ATCC1015 genomic DNA with the primers P1-P6 as described in Table 2. The *EgALase*, *MgGALDH* and the *hph* gene (from JKp1) were amplified with the primers P7-P12 (Table 2). All the six amplified fragments, containing 40 bp flanks for homologous recombination, and an *EcoRI* and *BamHI* digested platform plasmid pRS426 were transformed to yeast using the Gietz method [31] and the transformants were selected on SCD-URA plates. Several yeast colonies were collected and the resulting plasmid Bidir-EgALase-MgGALDH was rescued and sequenced with the primers P13-P25 (Table 2). The resulting expression cassette was linearized with *EcoRI* and *BamHI* (both NEB) before *A. niger* was transformed.

**Table 2:** **Primers used in this work**

**Primer**	**Sequence**	**Description**
P1	AGGCATCTGTCTGAGAGGCAACCGTGGCGA	Amplification of *gaaA/C* promoter, forward
	GAGTCCGCATTCTTTGATCTGCTGTTAGTT	
P2	GGTGACGAAGTGTGCGATTGAGCGTGATAA	Amplification of *gaaA/C* promoter, reverse
	AACGAAACATTGTGATTGCTGTGGTGTAAA	
P3	TAAGTTGGAGAAGTTGTTTCCGTCGCTCGAT	Amplification of *gaaA* terminator, forward
	GCCATTTGAATACCTTAGAGAAGCTTGTATG	
P4	CGTCTCTCCGCATGCCAGAAAGAGTCACCGG	Amplification of *gaaA* terminator, reverse
	TCACTGTACCATCTCCATCTCCTTCCCG	
P5	GCCCCCCCTCGAGGTCGACGGTATCGATAAGC	Amplification of *gaaC* terminator, forward
	TTGATATCGAATTCCTGTTGGAGAGAGGGTGTGT	
P6	CCCAGCCCCAGGTCCGCCACCCGCAGAGTTCCGT	Amplification of *gaaC* terminator, reverse
	TTGTGATCCATTGTATCATATAGATTATGAC	
P7	ATGCGGACTCTCGCCACG	Amplification of *Eg* ALase, forward
P8	TCACAAACGGAACTCTGCGG	Amplification of *Eg* ALase, reverse
P9	ATGTTTCGTTTTATCACGCT	Amplification of *Mg* GALDH, forward
P10	TCAAATGGCATCGAGCGAC	Amplification of *Mg* GALDH, reverse
P11	GTACAGTGACCGGTGACTCT	Amplification of *hph*, forward
P12	GCTGGAGCTCCACCGCGGTGGCGGCCGCTCTAGA	Amplification of *hph*, reverse
	ACTAGTGGATCCTTGGAGATTTCAGTAACGTT	
P13	GAGGTCGACGGTATCGATAAGC	Sequencing of the bidirectional *Eg*/*Mg* cassette
P14	TGATACAATGGATCACAAACGG	"
P15	CAACAGAGAACAGACCGCCA	"
P16	GTGTTGCGAAGCTGTAGTTGG	"
P17	ATTTACACCACAGCAATCAC	"
P18	AAAGAAGCGTGTTCGAGTCC	"
P19	ATACGGAGGATGAAGCCCTC	"
P20	GCCAGCGGAAGGAGATTACG	"
P21	GGCAGTGATTGAGGCTGTGG	"
P22	AGTAAGCGAAGGAGAATGTG	"
P23	AGTACTTTGCTACATCCATACTCC	"
P24	ATTCGGACCGCAAGGAATCG	"
P25	TGTCGGGCGTACACAAATCG	"
P26	AGCCGTGTTTCAATGTCGTG	"
P27	CGCTCTAGAACTAGTGGATC	"
P28	CAACATTGTCATGTCTGGTGG	qPCR of *actin*, forward
P29	GGAGGAGCAATGATCTTGAC	qPCR of *actin*, reverse
P30	AGGACACGATTACTCTACTTG	qPCR of *gaaA*, forward
P31	GAGCCCATATAATGGAAGTAC	qPCR of *gaaA*, reverse
P32	TCCGGGTGGACCCCGCTAAG	qPCR of *EgALase*, forward
P33	TGAAACACGGCTCCGGCGTC	qPCR of *EgALase*, reverse
P34	TAGCAAGTGGCGCGGTGTCC	qPCR of *MgGALDH*, forward
P35	TCGTGATCTCACCGCCCCGA	qPCR of *MgGALDH*, reverse

All the resulting expression cassettes were introduced into the *A. niger* Δ*gaaB* strain by protoplast transformation method [32]. Transformants were screened for integration of the cassette by growth in the presence of 400 μg ml^−1^ hygromycin B (Calbiochem). Strains having both genes (*smp30* or *EgALase* + *MgGALDH*) expressed in JKp1 under *gpdA* promoter were generated by co-transformation. Integration of the transformed cassettes into the genome was confirmed with colony PCR using Phire direct PCR kit (Thermo Scientific) and the primers P26 and P27 (Table 2).

### Media and culture conditions

Luria Broth (LB) culture medium supplemented with 100 μg ml^−1^ of ampicillin and cultural conditions of 37°C and 250 rpm were used for *E. coli* cultures. For yeast pre-cultures, YPD medium (10 g yeast extract l^−1^, 20 g peptone l^−1^ and 20 g D-glucose l^−1^) was used and after transformation SCD-URA plates (uracil deficient synthetic complete media supplemented with 20 g D-glucose l^−1^) were used for uracil auxotrophy selection. Yeast cultures were carried out at 30°C and all the liquid cultures at 250 rpm. For the *A. niger* submerged fermentations, spores were generated on potato-dextrose plates and 0.9*10^8^ spores were inoculated into 50 ml of YP medium (10 g yeast extract l^−1^, 20 g peptone l^−1^) containing 30 g gelatin l^−1^. Mycelia were pre-grown in 250-ml Erlenmeyer flasks by incubating overnight at 28°C, 200 rpm and harvested by vacuum filtration, rinsed with sterile water and weighted. In the *A. niger* transformations and fermentations, *A. nidulans* defined minimal medium [33], containing (in g l^−1^) 6 NaNO_3_, 0.52 KCl, 0.52 MgCl_2_ and 1.52 KH_2_PO_4_ was used. In the transformations the minimal medium was supplemented with 1.2 M D-sorbitol, 10 g l^−1^ of D-glucose and 20 g l^−1^ of agar and the pH was adjusted to 6.5. The minimal medium used in the fermentations for L-AA production, enzymatic assays and transcriptional analysis was supplemented with 20 g l^−1^ of D-galUA and 5 g l^−1^ of D-xylose or with 40 g l^−1^ of dried citrus processing waste (CPW). The pH was adjusted to 3. The CPW contained peel and pulp from the processed oranges and was received from the Federal University of Paraná in Curitiba, Brazil. The submerged cultures supplemented with pure D-galUA and D-xylose were inoculated with 1 g l^−1^ (cdw) and the submerged cultures supplemented with CPW were inoculated with 3 g l^−1^ (cdw) of pre-grown mycelia.

### Enzymatic assays

For enzymatic assays, *A. niger* mycelia were harvested by vacuum filtration. Crude extracts were prepared in 100 mM sodium phosphate buffer pH 7 with protease inhibitor (Complete, Roche). The cells were disrupted with 0.4 mm diameter glass beads using a bead beater (Precellys 24, Bertin Technologies) and solid residues were removed by centrifugation. The GALDH assay was based on the cytochrome c reduction at 550 nm as described by Leferink et al. [26]. Crude extracts from the each strain were supplemented with 500 μM of FAD (flavin adenine dinucleotide), which is an essential cofactor for MgGALDH, and were tested for the activity by following the cytochrome c reduction after addition of 5 mM L-galL. The enzymatic assay for the lactonase activity (from EgALase and Smp30) was based on a pH change in the reaction from L-galA to L-galL that absorbs protons and was assayed as described by Ishikawa et al. [16]. The pH was followed at 405 nm using p-nitrophenol as a pH indicator and the correspondence between ∆405 nm and the absorption of protons were quantified using the titration with HCl.

### Transcriptional analysis

Samples of 2 ml were collected from the cultures and the mycelium was harvested by vacuum filtration. The filtered mycelium was frozen with liquid nitrogen and stored at −80°C. RNA was extracted using the RNeasy Plant Mini Kit (Qiagen) and ~1 μg of total RNA was used for cDNA synthesis with the Transcriptor High Fidelity cDNA Synthesis Kit (Roche), following the manufacturer’s instructions. cDNA samples were diluted with RNase free water (Roche) and were used for RT-qPCR using a LightCycler II with the LightCycler SYBR green I Master mix (both Roche). The expression of *gaaA*, *EgALase*, *MgGALDH* and actin (ATCC 1015 200483-mRNA) were quantified using the primers P28-P35 (Table 2). The signal from each of the target genes was normalized to actin using the accompanying software (Advance Relative Quantification tool).

### Chemical analyses

Samples of 2 ml were removed at intervals and mycelium was separated from the supernatant by centrifugation. The concentration of D-galUA and L-galA was determined by HPLC using a Fast Acid Analysis Column (100 mm × 7.8 mm, BioRad Laboratories, Hercules, CA) linked to an Aminex HPX-87H organic acid analysis column (300 mm × 7.8 mm, BioRad Laboratories) with 5.0 mM H_2_SO_4_ as eluent and a flow rate of 0.5 ml min^−1^. The column was maintained at 55°C. Peaks were detected using a Waters 410 differential refractometer and a Waters 2487 dual wavelength UV (210 nm) detector. The L-AA content was measured using the commercial Ascorbic Acid Assay Kit II (Sigma-Aldrich) following the manufacturer’s instructions.

## Abbreviations

L-AA: L-ascorbic acid
D-EA: D-erythroascorbic acid
D-galUA: D-galacturonic acid
L-galA: L-galactonic acid
L-galL: L-galactono-1,4-lactone
GALDH: L-galactono-1,4-lactone dehydrogenase
ALase: Aldonolactonase
CPW: Citrus processing waste
